# Evidence for Long-Term Persistence and Local Diversification of the White-Tailed Sea-Eagle *Haliaeetus albicilla* in the Lower Danube Region: Insights from Mitochondrial DNA Phylogeography

**DOI:** 10.3390/genes17080933

**Published:** 2026-08-11

**Authors:** Mitică Ciorpac, Vasile Alexe, Alexandru-Cătălin Doroșencu, Lucian-Eugen Bolboacă, Janos Botond Kiss, Dumitru Murariu, Marian Tudor, Mihai Marinov

**Affiliations:** 1 Advanced Research and Development Center for Experimental Medicine (CEMEX), Grigore T. Popa University of Medicine and Pharmacy, 16 Universitatii Street, 700115 Iași, Romania; ciorpac.mitica@gmail.com; 2 Department of Biodiversity Conservation and Sustainable Use of Natural Resources, Danube Delta National Institute for Research and Development, 165 Babadag Street, 820112 Tulcea, Romania; alexandru.dorosencu@ddni.ro (A.-C.D.); marian.tudor@ddni.ro (M.T.); mihai.marinov@ddni.ro (M.M.); 3 Department of Center for the Study of Transborder and Emergent Diseases and Zoonoses, Danube Delta National Institute for Research and Development, 165 Babadag Street, 820112 Tulcea, Romania; lucian.bolboaca@ddni.ro; 4 Independent Researcher, 4 Frasinului Street, 820187 Tulcea, Romania; jbkiss03@yahoo.com; 5 Romanian Academy, Calea Victoriei Street, 127, 010962 Bucharest, Romania; dumitru.murariu@acad.ro

**Keywords:** *Haliaeetus albicilla*, Danube Delta Biosphere Reserve, phylogeography, mitochondrial DNA, genetic diversity, refugium, haplotype diversity

## Abstract

**Background/Objectives:** The White-tailed Sea-eagle, *Haliaeetus albicilla*, experienced severe population declines throughout Europe during the nineteenth and twentieth centuries, followed by a remarkable recovery in recent decades. Although the genetic structure of northern and central European populations has been extensively investigated, the phylogeographic history and contribution of the lower Danube population to the mitochondrial genetic diversity of the species remain poorly understood. **Methods:** We analyzed mitochondrial control region (MT-CR HVR1) sequences from White-tailed Sea-eagles breeding in the Danube Delta Biosphere Reserve (DDBR) and integrated these data with previously published sequences covering the species’ distribution across Europe and Asia; a total of 480 samples were examined. **Results:** The analysis revealed 47 haplotypes, including 11 haplotypes unique to the DDBR population. Phylogeographic analyses support the Scandinavian Peninsula as the ancestral source area of the species, followed by an early colonization of Central Europe and subsequent expansion into the Balkan Peninsula through the Danube corridor. The Romanian population exhibited the highest haplotype diversity among the investigated regions (Hd = 0.9367) and was characterized by numerous private haplotypes and a predominance of haplogroup C (approximately 76% of individuals), which was rare or absent in most northern and central European populations. Demographic analyses revealed significant evidence of recent population expansion following historical bottlenecks (Fu’s Fs = −5.736, *p* = 0.006), whereas gene-flow estimates indicated only moderate connectivity with neighbouring Balkan and Central European populations. **Conclusions:** The high frequency of private haplotypes, the absence of significant isolation-by-distance patterns, and the distinct distribution of mitochondrial haplogroups, characterized by the predominance of haplogroup C, suggest that the current DDBR population may have originated primarily through local demographic recovery rather than recent large-scale recolonization. Our results indicate that the lower Danube region represents an important reservoir of genetic diversity and support the hypothesis that the Danube Delta may have acted as a potential secondary refugium and center of diversification for *H. a.* in southeastern Europe.

## 1. Introduction

The White-tailed Sea-eagle *Haliaeetus albicilla* Linnaeus, 1758 is one of the largest avian predators in the Palearctic region and is widely recognized as both a flagship and umbrella species for wetland and riparian ecosystems throughout Europe [1]. Historically, the species occupied a vast distribution range extending from Greenland and Iceland across Europe and northern Asia to the Pacific coast of eastern Russia and Japan. Europe currently supports approximately 50–74% of the global breeding population, highlighting its central role in the long-term conservation of the species [2].

Throughout Europe, the White-tailed Sea-eagle experienced severe population declines during the nineteenth and twentieth centuries. Direct persecution, habitat destruction, reduction in old-growth forests suitable for nesting, environmental contamination by organochlorine compounds, and secondary poisoning resulted in the disappearance of numerous local populations and a dramatic contraction of the species’ range [3,4]. By the 1970s, several European populations had been reduced to only a few breeding pairs, while an estimated 70% of the remaining European population persisted along the Norwegian Atlantic coast [4,5].

The implementation of legal protection measures, restrictions on pesticide use, habitat conservation programs, and active management initiatives led to a remarkable recovery of White-tailed Sea-eagle populations throughout Europe during the last decades [6,7]. As a consequence, the species has recolonized many former breeding areas, and population expansion is currently occurring across large parts of northern, central, and eastern Europe. This recovery has generated considerable interest in understanding the evolutionary history, dispersal dynamics, and genetic structure of contemporary populations.

Previous molecular studies have revealed unexpectedly high levels of genetic diversity despite historical population bottlenecks and have demonstrated substantial phylogeographic structure across the species’ range [8,9,10,11,12,13]. These investigations identified Scandinavia as a major stronghold for the species and highlighted the importance of Central Europe in shaping contemporary genetic diversity. Furthermore, patterns of mitochondrial variation suggested postglacial expansion from northern refugial populations and subsequent colonization of adjacent regions [8,10].

However, a major gap remains in our understanding of White-tailed Sea-eagle phylogeography. Most previous studies focused on northern and central European populations, whereas populations inhabiting the lower Danube Basin and the Balkans have received comparatively little attention. Consequently, the evolutionary significance of the Danube populations, particularly those breeding in the Danube Delta Biosphere Reserve (DDBR), remains poorly understood.

The Danube Delta represents one of the largest and best-preserved wetlands in Europe and constitutes an important breeding area for the White-tailed Sea-eagle in southeastern Europe. Historical records indicate that the species was once abundant in the region [14], followed by dramatic declines caused by persecution, habitat alteration, poisoning campaigns, and environmental contamination during the twentieth century [15,16,17,18]. Since the establishment of the Danube Delta Biosphere Reserve in 1990 and the implementation of conservation measures, the population has undergone a substantial recovery, increasing from fewer than 10 breeding pairs during the 1980s to approximately 80 occupied nests in recent years [18].

This remarkable demographic recovery raises an important evolutionary question: does the contemporary Danube Delta population represent a recent recolonization event driven by immigrants from northern and central European populations, or does it primarily reflect the demographic resurgence of a historically persistent local lineage? Resolving this question is essential for understanding the evolutionary history of the species in southeastern Europe and for establishing appropriate conservation priorities.

The present study aims to investigate the phylogeographic origin, genetic diversity, and demographic history of White-tailed Sea-eagles breeding in the Danube Delta Biosphere Reserve and surrounding areas. Using mitochondrial control region sequences and a comparative dataset covering the species’ distribution across Europe and Asia, we reconstruct colonization pathways, evaluate patterns of gene flow, and test whether the lower Danube region represents merely a recolonized peripheral population or an important center of long-term persistence and diversification for *H.a*.

## 2. Materials and Methods

### 2.1. Study Site

The nest search method entailed navigating the canals and lakes of the DDBR in Romania. Identification of White-tailed Sea-eagle nests was conducted in two stages. The first stage occurred during the winter–pre-spring season (December to March), when the trees lacked foliage, enabling easy visibility of nests from several kilometers away. The second stage occurred during the pre-spring–spring season (April to June) to evaluate breeding success. When an adult bird was spotted on a tree, the observer approached by boat and searched for the nest within a 300-m radius. Typically, the adult bird, usually the male, would remain close to the nest, serving as a sentinel. A nest was considered occupied if a bird was observed on or near it during the breeding season or if chicks were present. Some feathers were also collected from the feeding areas of the white-tailed eagle near known nests.

### 2.2. Sampling, DNA Amplification and Sequencing

**Figure 1:**
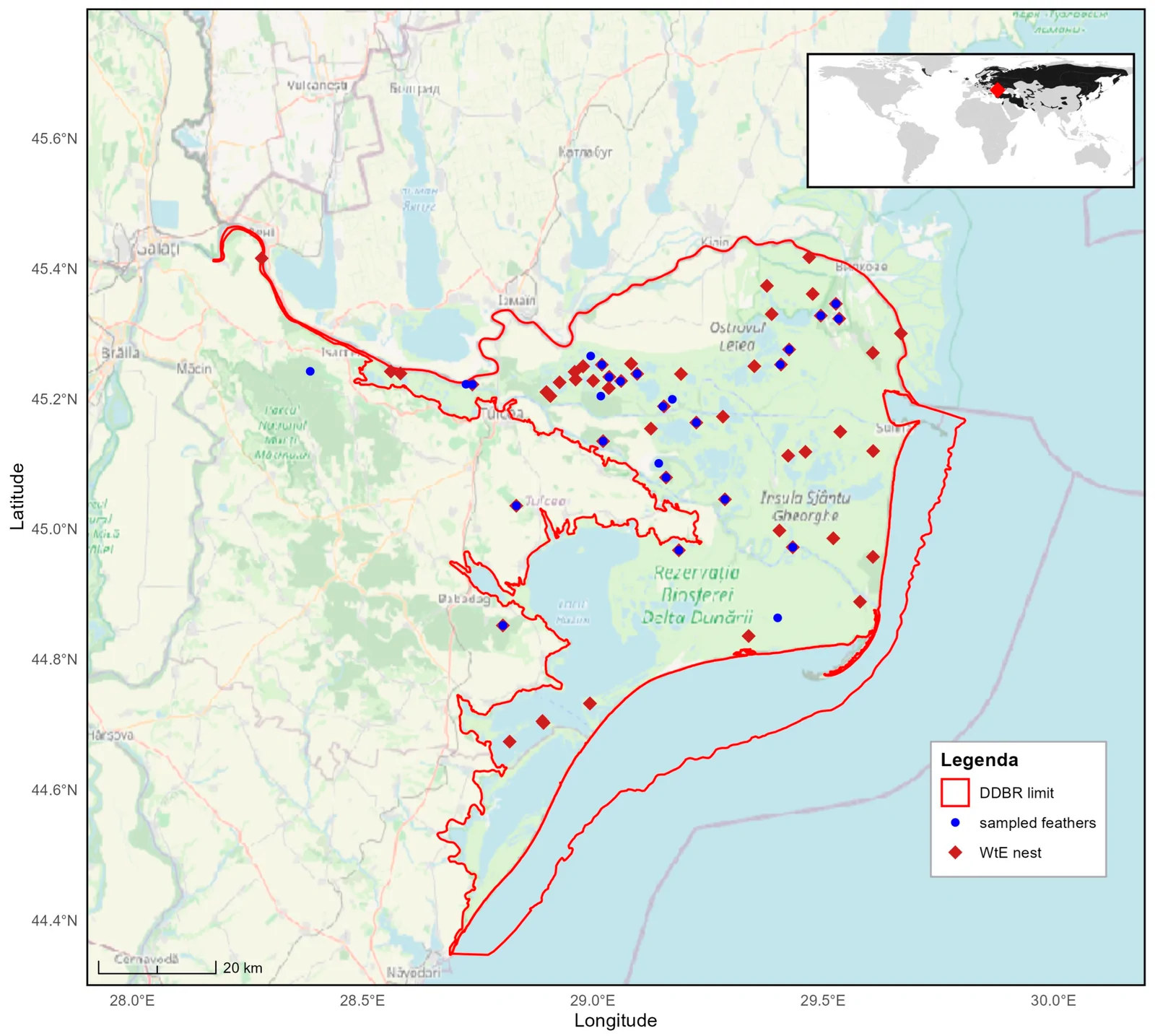
Geographical distribution of feather samples collected and nests used by individuals of *H. a.* in DDBR and its surrounding areas.

Genetic diversity and population structure analysis utilized biological material obtained from nests within the DDBR and its environs. Sampling took place over two years, 2017–2018, within the DDBR and adjacent areas (see Figure 1). Approximately 56 breeding pairs were identified during this period, and 27 feather samples were collected from 26 nests, representing approximately 50% of the occupied nests. Access to the remaining nests was not possible, because most of them were located in marshy areas with limited or no accessibility. Biological material was collected non-invasively and consisted of naturally moulted feathers found after the breeding period in the vicinity of occupied nests and at known feeding or perching sites. During field sampling, preference was given to large feathers, particularly flight and tail feathers, because their larger calami generally contain more host DNA and provide a higher likelihood of successful amplification than small body feathers. Small feathers were therefore generally not selected for molecular analysis. For each sample, DNA was extracted from the calamus of a single feather, and feathers were never pooled. This procedure minimized the possibility of mixed DNA from multiple individuals within a single extraction. However, because feathers were collected non-invasively, their origin could not always be assigned with certainty to an adult or a chick, and the possibility that separate feathers originated from the same individual cannot be completely excluded. The shaft fragment of the collected feathers underwent genomic DNA extraction using the NucleoSpin^®^ kit (MACHEREY-NAGEL GmbH & Co. KG, Düren, Germany), with an overnight enzymatic digestion step using lysis buffer and Proteinase K (10 mg/mL). Subsequent extraction steps were conducted according to the manufacturer’s specifications. Amplification of the mitochondrial control region (MT-CR HVR1) was performed using the HalHVR1 primer pair previously developed by Hailer et al. [8]. The primers targeted a 544-bp region spanning domains I and II, which contains most of the mitochondrial control-region variability. Sequencing of the amplicons was performed using the Dye-Terminator method through a commercial sequencing service (Macrogen Inc. (Macrogen Europe B.V., Amsterdam, The Netherlands).). Raw sequencing chromatograms were visually inspected, assembled, and manually verified using DNABaser (Heracle BioSoft SRL, Mioveni, Romania.). Base calls generated automatically during sequence processing were checked against the original chromatograms, and ambiguous nucleotide calls, which were infrequent, were manually inspected and corrected only when clearly supported by the electropherogram. Low-quality terminal regions were trimmed prior to sequence alignment. The edited sequences were exported in FASTA format and aligned using the ClustalW algorithm implemented in MEGA X [19]. The resulting HVR1 sequences were 498 bp in length after trimming. From 27 extracted DNA samples, 25 were successfully amplified, while two samples failed due to insufficient DNA yield.

### 2.3. Genetic Diversity

To facilitate a comprehensive phylogeographic analysis, we compiled a dataset comprising sequences of the mitochondrial control region obtained in this study (N = 25), as well as those obtained by Hailer et al. [8], Honnen et al. [9] and Langguth et al. [10]. Combining these datasets yielded a total of 480 individuals analyzed, representing more than 15 countries across Europe. These countries include the northern part (N = 185, Iceland and the Scandinavian peninsula), Central and Eastern Europe (N = 183), Southeastern Europe—the Balkan Peninsula (N = 32, Greece, Serbia), Romania (N = 25), and Central & Far East Asia (N = 55, Kazakhstan, Amur, and Japan). A summary of the mitochondrial control-region dataset used in the comparative analyses, including the geographic populations, source publications, number of sequences contributed by each study, and the corresponding GenBank accession number ranges for the published datasets, is provided in Supplementary Table S1. Alignment of the newly generated sequences with the published dataset produced a final alignment of 498 bp, which was used for all subsequent analyses. All sequences underwent analysis for haplotypes, genetic diversity parameters (Hd—haplotype diversity, K—average number of differences, He—expected heterozygosity for polymorphic loci, π—nucleotide diversity), and selective neutrality tests (Tajima’s selective neutrality test, Chakraborty’s amalgamation test, and Fu’s neutrality test) using ARLEQUIN v.3.5 [20]. Standard molecular indexes of differentiation and gene flow were calculated using DNASP v.5.10 [21] to quantify the level of genetic differentiation. Differentiation was expressed as the average number of nucleotide differences between populations (Kxy), the average number of nucleotide substitutions per site between populations (Dxy), and the net number of nucleotide substitutions per site between populations (Da). Gene flow estimates were computed from haplotype data information, including the genetic differentiation index based on the frequency of haplotypes (Gst) and gene flow (Nm), as well as from nucleotide sequence data information, such as Wright’s F-statistics (Fst) and the proportion of interpopulational gene diversity (γst). To illustrate the relationship between haplotypes within all populations, the TempNet script [22], run in R [23], was employed to construct a statistical parsimony network. For descriptive purposes, haplotypes occupying central positions and exhibiting high frequencies within the statistical parsimony network were considered putatively ancestral, following the commonly accepted interpretation that centrally connected haplotypes are more likely to represent older maternal lineages. No explicit ancestral-state reconstruction was performed. The three major haplogroups (A–C) were identified as clusters of closely related haplotypes separated by multiple mutational steps within the network and were used as descriptive units for interpreting the observed phylogeographic patterns.

## 3. Results

### 3.1. Mitochondrial Diversity and Haplotype Distribution

A total of 480 White-tailed Sea-eagle mitochondrial control-region (HVR1) sequences, each 498 bp in length, were included in the comparative analyses, representing populations from northern Europe, Central Europe, the Balkan Peninsula, Romania, and Asia. The aligned dataset contained 34 variable (segregating) sites, which defined 47 distinct haplotypes across all populations, of which 11 were exclusive to the Danube Delta Biosphere Reserve (DDBR) population.

**Figure 2:**
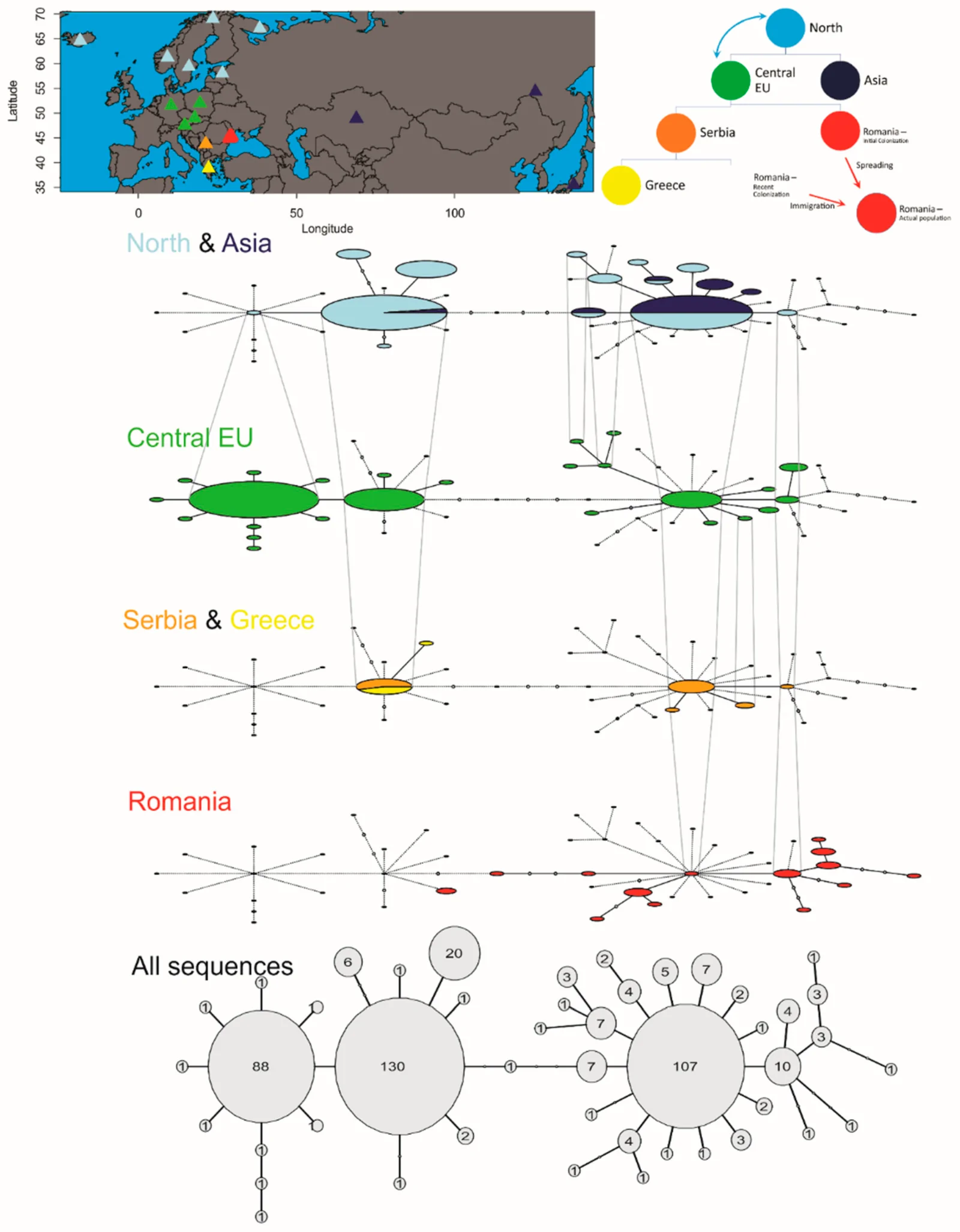
The phylogeographic origin of Haliaeetus albicilla populations (**top left**). Geographical distribution of the samples used in the analysis; (**top right**) schematic representation of the colonization pattern; (**bottom**) haplotype network and the absolute frequency for the populations: Nordic, Asian, Central European, Serbia, Greece, and Romania. Node size is proportional to haplotype frequency, and small black points along connecting branches represent individual mutational steps. Centrally positioned, high-frequency, and geographically widespread haplotypes were interpreted as putatively ancestral.

The geographic distribution of haplotypes revealed clear phylogeographic structuring (Figure 2). Individuals from Asia predominantly possessed haplotypes shared with Scandinavian populations, suggesting a putatively ancestral eastward expansion originating from northern Europe. However, the presence of several region-specific Asian haplotypes is consistent with subsequent local diversification following colonization.

The Scandinavian population retained the highest frequencies of putatively ancestral haplotypes, identified as the centrally positioned and most widely distributed haplotypes in the statistical parsimony network. In contrast, Central European populations displayed greater haplotypic complexity, including the emergence of a distinct putatively ancestral haplotype and several derived lineages. This pattern is consistent within Central Europe, and may have acted as the first major diversification centre following the initial expansion from Scandinavia.

The Balkan populations (Serbia and Greece) contained a mixture of widespread European haplotypes and a limited number of private haplotypes. The occurrence of region-specific haplotypes at low frequencies suggests the persistence of localized maternal lineages despite substantial historical gene flow.

The Romanian population differed markedly from all other populations by harboring 78.6% private haplotypes and exhibiting a distinctive haplotype composition. Of the 14 haplotypes detected in the Romanian population, 11 were private to the Danube Delta Biosphere Reserve, while only three haplotypes, represented by six individuals, were shared with other European populations. Shared European haplotypes occurred at relatively low frequencies, whereas locally derived haplotypes were well represented, indicating long-term persistence of maternal lineages within the lower Danube region.

### 3.2. Genetic Diversity

Genetic diversity indices varied considerably among populations (Table 1). The Romanian population exhibited the highest haplotype diversity recorded among all investigated regions (Hd = 0.9367 ± 0.0316), despite a relatively moderate sample size. Fourteen haplotypes and sixteen polymorphic sites were identified within the Romanian dataset, demonstrating substantial mitochondrial variability.

Comparable levels of diversity were observed only in Murmansk (Hd = 0.9333) and the Czech Republic (Hd = 0.8952), whereas several populations from Central Europe exhibited considerably lower diversity values. The Scandinavian populations generally showed moderate to high diversity but were dominated by a smaller number of putatively ancestral haplotypes.

Nucleotide diversity in Romania (π = 0.00679) was among the highest recorded in the study, further supporting the existence of a genetically diverse and evolutionarily important population.

Neutrality tests revealed signals consistent with demographic expansion in several populations. The strongest signal was observed in Romania, where Fu’s Fs was significantly negative (Fs = −5.736, *p* = 0.006). This pattern is consistent with a recent population expansion following a historical bottleneck, although significant neutrality statistics may also result from selective processes. Tajima’s D values were generally negative but not statistically significant. Therefore, the demographic interpretation is based on the combined evidence from Fu’s Fs, the star-like haplotype network, and the mismatch distribution, rather than on Tajima’s D alone.

### 3.3. Haplotype Network and Phylogeographic Relationships

The haplotype network revealed a hierarchical structure consistent with the species’ evolutionary history (Figure 2). The statistical parsimony network was dominated by several high-frequency, centrally positioned haplotypes that were widely distributed across geographic regions and were therefore interpreted as putatively ancestral. Northern European samples were concentrated mainly in a small number of high-frequency central haplotypes with relatively few low-frequency derivatives. In contrast, the pooled Central European samples were distributed among a larger number of intermediate- and low-frequency haplotypes across several connected branches.

Scandinavian haplotypes occupied central positions within the statistical parsimony network and formed the principal cluster of putatively ancestral haplotypes, from which most European and Asian haplotypes were connected by relatively few mutational steps.

Central European populations occupied an intermediate position and contained both putatively ancestral Scandinavian haplotypes and newly derived lineages. The network topology supports the hypothesis that Central Europe represented the first major colonization area following expansion from Scandinavia.

The Balkan populations formed peripheral clusters connected primarily through Central European haplotypes, supporting a southward expansion through the Danube Basin.

The Romanian population displayed a distinct pattern. Of the 14 haplotypes detected in Romania, 11 were private to the DDBR and three were shared with other European populations. The three shared haplotypes were represented by six of the 25 Romanian individuals (24%), whereas 19 individuals (76%) carried private haplotypes. All private Romanian haplotypes occupied terminal positions in the network and differed from the nearest shared haplotypes by no more than two mutational steps. The presence of multiple endemic haplotypes with moderate frequencies suggests local diversification rather than recent founder events.

### 3.4. Haplogroup Structure

**Figure 3:**
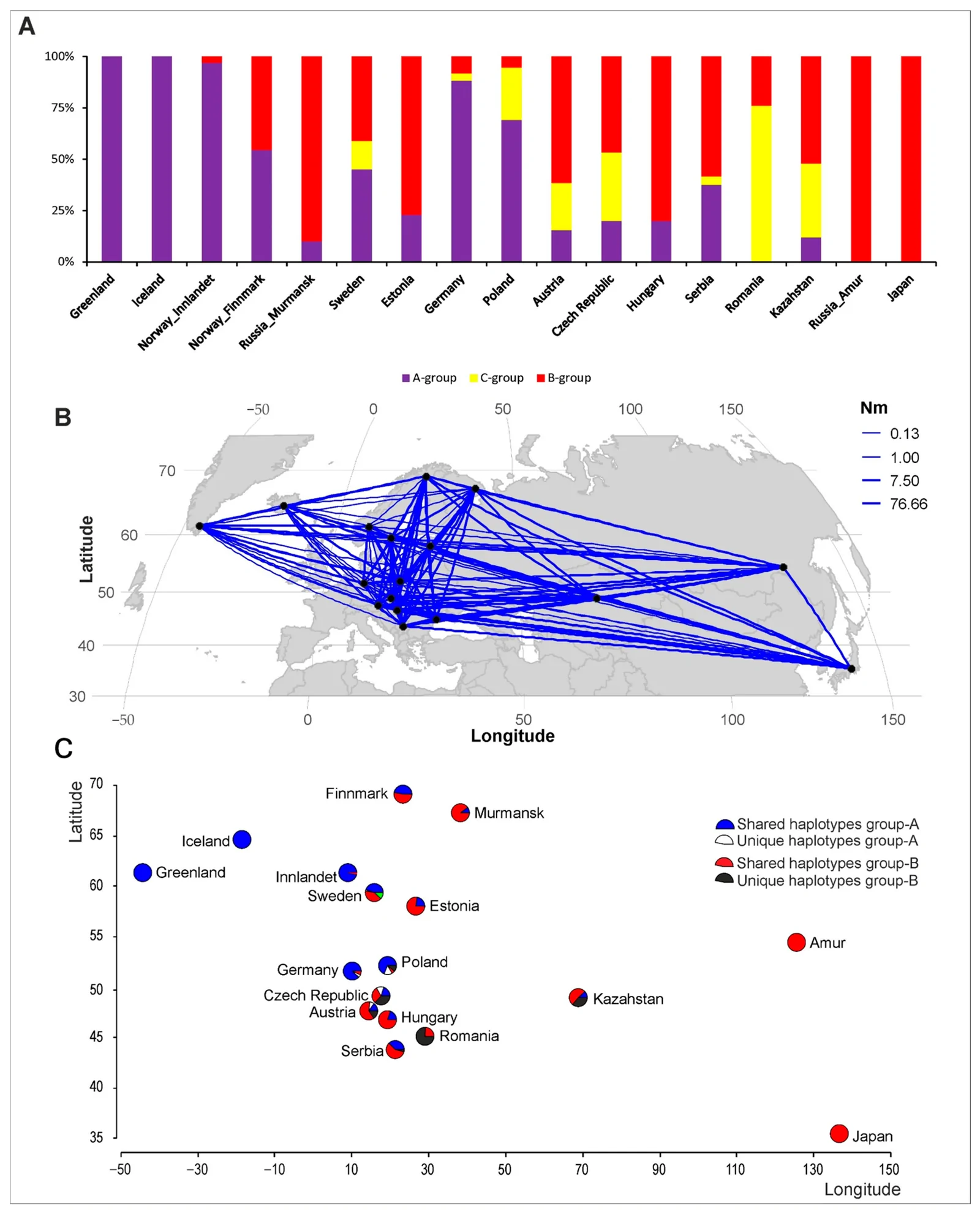
Phylogeographic structure and population connectivity of White-tailed Sea-eagles across Europe and Asia based on mitochondrial control region (HVR1) sequences. (**A**) Relative frequencies of the three mitochondrial haplogroups (**A**–**C**) identified in the studied populations. Northern populations (Greenland, Iceland, Norway, Sweden, and Finland) are predominantly characterized by haplogroup A, whereas Central European populations exhibit a mixture of haplogroups A and B. In contrast, the Romanian population from the Danube Delta Biosphere Reserve is dominated by haplogroup C, indicating a distinct maternal lineage and a unique phylogeographic history within southeastern Europe. (**B**) Gene-flow network among populations inferred from Nm estimates. Line thickness is proportional to the magnitude of gene flow. The network highlights historical and contemporary genetic connectivity among European populations, particularly along the Central European–Danube corridor. (**C**) Geographic distribution of shared and unique haplotypes belonging to the major mitochondrial haplogroups. Circles represent shared haplotypes, whereas triangles indicate population-specific haplotypes. The occurrence of numerous unique haplotypes in the Danube Delta Biosphere Reserve supports long-term persistence and local diversification of the species in the lower Danube region.

Three major mitochondrial haplogroups were identified across the study area (Figure 3A). Northern European populations were overwhelmingly dominated by haplogroup A, which represented the ancestral Scandinavian lineage. Central European populations exhibited a mixed composition, with haplogroups A and B occurring at relatively similar frequencies.

In contrast, the Romanian population displayed a markedly different structure. Haplogroup C accounted for approximately 76% of all individuals, whereas haplogroup B represented approximately 24%. Haplogroup A was absent from the Romanian samples. The predominance of haplogroup C distinguishes the DDBR population from northern and central European populations and suggests the existence of an independent evolutionary lineage associated with the lower Danube region.

Serbian and Greek populations showed intermediate patterns characterized by mixed haplogroup composition but lacked the strong dominance of haplogroup C observed in Romania.

### 3.5. Gene Flow and Population Connectivity

Gene-flow estimates revealed substantial variation among populations (Figure 3B,C). The Romanian population showed the strongest genetic connectivity with populations from Central Europe, particularly the Czech Republic (Nm = 5.18), Murmansk (Nm = 4.60), Hungary (Nm = 4.16), Serbia (Nm = 2.78) and Austria (Nm = 3.74).

Moderate connectivity was observed between Romania and Serbia (Nm = 2.78), suggesting the existence of historical dispersal routes along the Danube corridor. Despite these relatively high levels of gene flow, the Romanian population retained a large number of private haplotypes and a unique haplogroup composition. This indicates that migration has not been sufficient to homogenize the mitochondrial gene pool of the DDBR population.

### 3.6. Exploratory Analysis of Spatial Genetic Structure

**Figure 4:**
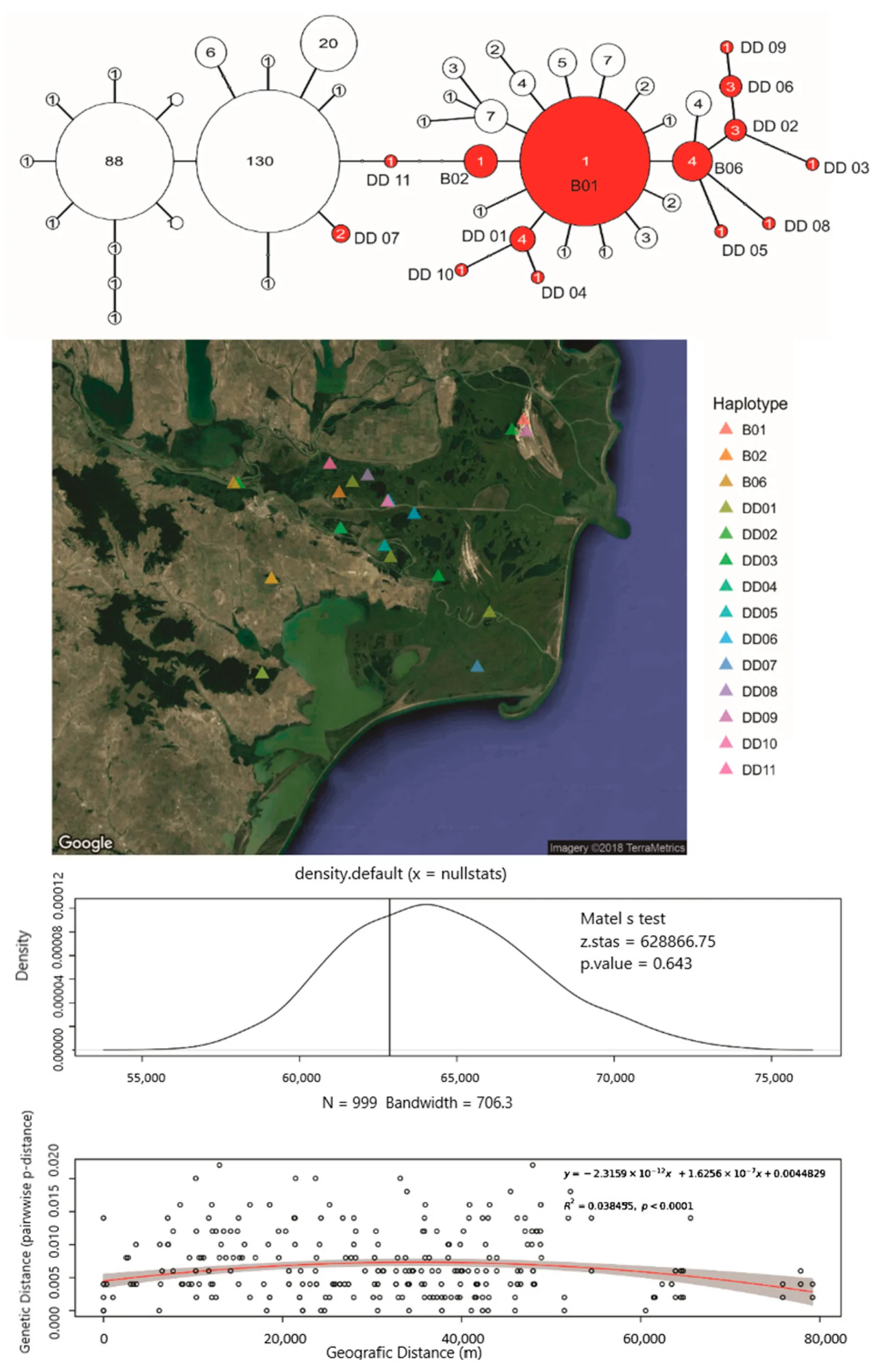
Phylogenetic relationships among mitochondrial haplotypes from the Danube Delta Biosphere Reserve (DDBR) and surrounding areas, their geographic distribution, and exploratory assessment of spatial genetic structure. In the haplotype network (upper panel), red nodes indicate haplotypes detected in the Romanian (DDBR) samples, whereas white nodes represent haplotypes from the comparative dataset; node size is proportional to haplotype frequency, and small circles along connecting branches indicate mutational steps. The map shows the geographic distribution of the 14 haplotypes detected within the Romanian dataset, with colors corresponding to individual haplotypes. The lower panels show the Mantel test and the relationship between pairwise genetic and geographic distances. No significant correlation between genetic and geographic distances was detected (*p* = 0.643).

The Mantel test did not detect a significant correlation between geographic and mitochondrial genetic distances among the Romanian samples (*p* = 0.643; Figure 4). Thus, no evidence of isolation by distance was identified within the limits of the present dataset. Given the relatively small sample size and the use of a single maternally inherited mitochondrial marker, this interpretation of the results is limited.

The absence of a significant correlation does not demonstrate that a fine-scale spatial genetic structure is absent, nor does it allow alternative demographic scenarios, including progressive recolonization, to be excluded. Rather, the analysis indicates that no clear spatial pattern was detectable with the current sampling design and marker resolution. Additional sampling and genome-wide nuclear data will be required to assess local genetic structure within the Danube Delta more robustly.

## 4. Discussion

The present study provides the first comprehensive phylogeographic assessment of the White-tailed Sea-eagle population inhabiting the DDBR and surrounding areas within a broad Eurasian context. By integrating newly generated mitochondrial DNA sequences with previously published datasets [24,25,26] from Europe and Asia, we demonstrate that the lower Danube population could represent a genetically distinct component of the species’ distribution range. The high mitochondrial genetic diversity observed in Romania, together with the presence of numerous private haplotypes and a distinctive distribution of mitochondrial haplogroups, suggests a more complex phylogeographic history than previously recognized.

Overall, the mitochondrial patterns observed in this study are consistent with a phylogeographic scenario involving multiple stages of postglacial expansion and regional diversification. Previous studies identified Scandinavia as the principal stronghold of the species during historical population declines and proposed northern Europe as the primary source of post-bottleneck recolonization across much of the continent [8,9,10]. Our results broadly support this hypothesis. The central position of Scandinavian haplotypes within the statistical parsimony network and their widespread occurrence across Eurasia are consistent with northern Europe representing an important source region for subsequent range expansion. The predominance of these haplotypes in Asian populations further supports an eastward expansion followed by local diversification, a pattern previously reported for several widespread Palearctic bird species.

However, the mitochondrial data also indicate that Scandinavian populations alone cannot fully explain the present-day distribution of genetic diversity. The elevated diversity detected in Central Europe, together with the occurrence of additional putatively ancestral haplotypes and numerous derived lineages, suggests that this region functioned not only as a dispersal corridor but also as an important centre of mitochondrial diversification. The observed haplotype distribution is further consistent with southward dispersal through the Danube corridor into southeastern Europe. Within this framework, the Danube Delta population may have originated from an early Balkan–Central European colonization event, followed by prolonged local persistence and subsequent mitochondrial diversification in the lower Danube region.

One of the most striking findings of the present study is the exceptionally high genetic diversity observed in the Romanian population. The DDBR population exhibited the highest haplotype diversity among all populations included in the analysis (Hd = 0.9367), despite its relatively restricted geographic range. Such levels of diversity are difficult to reconcile with a recolonization scenario and are more consistent with the persistence of a substantial local maternal gene pool. The identification of 11 private mitochondrial haplotypes in the DDBR is consistent with this interpretation. Endemic haplotypes typically arise through long-term local evolution and are unlikely to accumulate rapidly following recent colonization events. Their moderate frequencies could indicate that these lineages are not recent mutations but rather components of a historically established population. Similar patterns have been reported in refugial populations of other avian species that persisted through periods of demographic contraction and subsequently expanded when environmental conditions improved.

Additional evidence for long-term persistence is provided by the haplogroup analysis. Whereas northern European populations were dominated by haplogroup A and Central European populations displayed a mixture of haplogroups A and B, the Romanian population was overwhelmingly characterized by haplogroup C. The dominance of this lineage, accounting for approximately three-quarters of all Romanian individuals, clearly distinguishes the DDBR population from the remainder of the species’ European range. Such a marked difference in haplogroup composition would be difficult to explain solely through recent immigration and instead could suggest an independent evolutionary trajectory in the lower Danube region.

The demographic analyses are consistent with this hypothesis. The significantly negative Fu’s Fs value could indicate a recent demographic expansion following a period of reduced population size. Historical records from the Danube Delta provide a compelling explanation for this pattern. During the nineteenth and twentieth centuries, White-tailed Sea-eagles experienced severe declines caused by direct persecution, habitat alteration, poisoning campaigns, and environmental contamination [15,16,17]. The subsequent establishment of the Danube Delta Biosphere Reserve, combined with legal protection measures and the reduction in environmental contaminants, facilitated a rapid demographic recovery. Therefore, the genetic signature observed today is consistent with a population that survived a bottleneck locally and subsequently expanded.

Gene-flow analyses revealed moderate genetic connectivity between Romania and neighbouring populations from Serbia, Hungary, Austria, and Central Europe. These results indicate that dispersal along the Danube corridor continues to occur and likely contributes to maintaining regional connectivity. Nevertheless, migration rates appear insufficient to erase the distinctive genetic signature of the DDBR population. If the current Romanian population had originated primarily through recent recolonization from neighbouring populations, one would expect a much greater representation of widespread Central European haplotypes and a substantially lower proportion of endemic lineages.

The absence of significant isolation-by-distance patterns provides additional support for this interpretation. Range expansions driven by progressive recolonization typically generate spatial gradients in genetic diversity, as neighbouring habitats are colonized sequentially. Such patterns were not observed in the present study. Instead, genetic variation appears largely independent of geographic distance, suggesting that the observed mitochondrial diversity is more consistent with local demographic recovery than a recent colonization wave.

Collectively, these mitochondrial patterns are consistent with a phylogeographic scenario in which the lower Danube region functioned as an important center of persistence and diversification for *H. a*. Although Scandinavian populations undoubtedly played a major role in the evolutionary history of the species, the Danube Delta appears to have retained a distinct maternal lineage through historical demographic fluctuations. The combination of high genetic diversity, numerous endemic haplotypes, dominance of haplogroup C, and signals consistent with local demographic expansion suggests that the DDBR population may represent more than a recently established peripheral population. From a conservation perspective, these results have important implications. The Danube Delta population harbors a disproportionately large fraction of the species’ mitochondrial diversity and may represent a unique evolutionary lineage within southeastern Europe. Consequently, conservation measures should prioritize not only population size and breeding success but also the preservation of genetic diversity and evolutionary processes. Although our mitochondrial results highlight the distinctiveness of the DDBR population, the available evidence based solely on a single mitochondrial marker is insufficient to formally designate it as an Evolutionarily Significant Unit (ESU). Nevertheless, the observed mitochondrial diversity suggests that this population may represent an important management unit whose status should be evaluated using genome-wide nuclear markers.

Overall, our findings challenge the traditional view that contemporary White-tailed Sea-eagle populations in southeastern Europe may have originated primarily through recolonization from northern populations. Instead, they support a more complex evolutionary scenario involving historical persistence, local diversification, demographic contraction, and subsequent recovery in the lower Danube region. This perspective highlights the importance of southeastern Europe as a reservoir of biodiversity and evolutionary history for one of the continent’s most iconic avian predators.

### Limitations

Several limitations should be considered when interpreting our results. Our inferences rely on a single, maternally inherited mitochondrial marker (the HVR1 control region), which reflects only the matrilineal component of population history and, over a relatively short and low-variability fragment, offers a limited resolution of fine-scale structure. The focal Danube Delta sample was modest (27 individuals from 26 nests, of which 25 sequences were analysed), and per-population sample sizes across the pan-European and Asian comparative dataset—compiled from previously published sequences and markedly uneven (8–85 individuals)—were sufficient to affect diversity estimates, network topology and neutrality statistics; the identification of the Romanian population as the most diverse and the signal of recent expansion should therefore be treated as provisional and confirmed with denser, more balanced sampling. Finally, in the absence of molecular dating and explicit coalescent modelling, the reconstructed scenario—including the role of the lower Danube as a potential secondary refugium—remains a working hypothesis that alternative explanations such as incomplete lineage sorting or secondary contact cannot yet formally exclude, and that future studies integrating genome-wide nuclear markers and explicit demographic modelling would allow to be tested directly.

## 5. Conclusions

This study provides the first comprehensive phylogeographic assessment of the White-tailed Sea-eagle population from the DDBR and surrounding areas within a broad Eurasian context. Mitochondrial DNA analyses revealed exceptionally high genetic diversity in the Romanian population, including the highest haplotype diversity recorded among all investigated populations and the presence of 11 private haplotypes. The predominance of haplogroup C, combined with the occurrence of numerous endemic maternal lineages, distinguishes the Danube Delta population from other European populations and is consistent with a unique evolutionary history within southeastern Europe. Consequently, demographic statistics such as Tajima’s D and Fu’s Fs could be interpreted as indicators of potential demographic trends.

The mitochondrial phylogeographic patterns identified in this study are consistent with a colonization scenario involving a putative Scandinavian source population, subsequent diversification in Central Europe, and southward expansion through the Danube corridor. The high frequency of private haplotypes, a signal consistent with post-bottleneck demographic expansion, and the absence of isolation-by-distance patterns suggest that the contemporary Danube Delta population may not be solely the result of recent recolonization. Rather, these mitochondrial data are consistent with the hypothesis that the lower Danube region has served as an area of long-term persistence that has contributed to the contemporary genetic diversity of the species.

The mitochondrial diversity observed in the Danube Delta population highlights its potential conservation importance within southeastern Europe. Future studies based on larger sample sizes and genome-wide nuclear markers will be essential to determine whether the distinct mitochondrial patterns identified here are also reflected in the nuclear genome and to further evaluate the conservation significance of this population.

## Figures and Tables

**Table 1:** Genetic diversity indices and neutrality-test statistics for White-tailed Sea-eagle populations from Europe and Asia inferred from mitochondrial control region (HVR1) sequences.

Pop.	N	Haps	S	Hd	K	He	π	Tajima’s D	Tajima’s D *p*-Value	Fu’s Fs	Fs *p*-Value	Pr (K ≥ k Obs)
Greece	8	2	1	0.25	0.25	0.25	0.00050	−105.482	0.216	−0.1819	0.207	0.45266
Iceland	26	2	1	0.41	0.41	0.41	0.00082	0.91864	0.875	118.111	0.594	0.83406
Norway Innlandet	33	2	6	0.06	0.36	0.06	0.00072	−210.255	0.001	109.641	0.54	0.17649
Norway Finn mark	22	6	8	0.67	3.43	0.43	0.00687	184.591	0.963	154.986	0.803	0.29295
Murmansk Russia	10	7	9	0.93	2.53	0.28	0.00507	−0.89195	0.221	−253.66	0.038	0.80258
Sweden	51	5	10	0.64	3.32	0.33	0.00665	140.976	0.933	459.542	0.947	0.68752
Estonia	35	5	7	0.78	2.38	0.34	0.00476	0.80484	0.831	144.082	0.781	0.87361
Germany	85	6	9	0.49	1.37	0.15	0.00275	−0.59882	0.342	0.49214	0.638	0.18172
Poland	55	15	19	0.74	2.77	0.14	0.00555	−10.656	0.158	−4.265	0.051	0.00273
Austria	13	7	10	0.77	2.59	0.26	0.00519	−0.89512	0.188	−177.63	0.099	0.15458
Czech Rep	15	8	11	0.90	4.42	0.40	0.00885	11.804	0.879	−0.5334	0.383	0.713
Hungary	15	4	8	0.7905	32.57	0.79	0.00652	11.926	0.356	27851	0.298	0.94187
Serbia	24	5	9	0.67	3.26	0.36	0.00653	115.702	0.865	264.426	0.881	0.55148
Romania	25	14	16	0.94	3.39	0.21	0.00679	−0.70563	0.266	−573.61	0.006	0.65136
Greece	8	2	1	0.25	0.25	0.25	0.00050	−105.482	0.216	−0.1819	0.207	0.45266
Kazakhstan	25	4	7	0.66	0.66	0.26	0.00371	−0.00084	0.569	19.035	0.852	0.76434
Amur Russia	22	3	2	0.32	0.34	0.17	0.00067	−0.87125	0.192	−0.8697	0.169	0.31774
Japan	8	2	1	0.25	0.25	0.25	0.00050	−105.482	0.207	−0.1819	0.209	0.45266

Parameters include sample size (N), number of haplotypes (haps), number of polymorphic sites (S), haplotype diversity (Hd), average number of nucleotide differences (K), expected heterozygosity (He), nucleotide diversity (π), Tajima’s D, Fu’s Fs, and Chakraborty’s amalgamation test. Among all investigated populations, the Danube Delta Biosphere Reserve population (Romania) displayed the highest haplotype diversity (Hd = 0.9367) and a significantly negative Fu’s Fs value (Fs = −5.736, *p* = 0.006), indicating a recent demographic expansion following a historical population contraction.

## Data Availability

The mitochondrial control-region sequences generated in this study have not yet been deposited in NCBI GenBank. The sequences will be submitted to GenBank prior to publication, and the corresponding accession numbers will be added to the manuscript once available. Until then, the sequences are available from the corresponding author upon reasonable request.

## Supplementary Materials

The following supporting information can be downloaded at: https://www.mdpi.com/article/10.3390/genes17080933/s1, Supplementary Table S1. Summary of the mitochondrial control-region (HVR1) sequences included in the comparative phylogeographic analyses of *H.a.*, indicating the geographic populations, source publications, number of sequences contributed by each study, and GenBank accession number ranges for the published datasets.
