# Supplementary material for: Evidence for Long-Term Persistence and Local Diversification of the White-Tailed Sea-Eagle Haliaeetus albicilla in the Lower Danube Region: Insights from Mitochondrial DNA Phylogeography
Source: Genes (Basel). 2026 Aug 11;17(8):933. doi: 10.3390/genes17080933 (PMC13512460; doi:10.3390/genes17080933)
Supplement: Supplementary file 1 [file genes-17-00933-s001.zip › genes-4458853-supplementary.pdf]

Supplementary Table S1. *Summary of the mitochondrial control-region (HVR1) sequences included in the comparative phylogeographic analyses of Haliaeetus albicilla, indicating the geographic populations, source publications, number of sequences contributed by each study, and GenBank accession number ranges for the published datasets.*

**Population/Study**	**Hailer et al., 2006**	**Hailer et al., 2007**	**Honnen et al., 2010**	**Langguth et al., 2012**	**Nemeshazy et al., 2013**	**Present study**	**Sum**
Greenland	-	8	-	-	-	-	8
Iceland	-	26	-	-	-	-	26
Norway (Innlandet)	33	-	-	-	-	-	33
Norway (Finnmark)	22	-	-	-	-	-	22
Russia (Murmansk)	10	-	-	-	-	-	10
Sweden	44	-	7	-	-	-	51
Estonia	12	-	-	23	-	-	35
Germany	18	-	67	-	-	-	85
Poland	-	-	-	55	-	-	55
Austria	-	-	13	-	-	-	13
Czech Republic	-	-	15	-	-	-	15
Hungary	-	-	-	-	15	-	15
Romania	-	-	-	-	-	25	25
Serbia	-	-	-	24	-	-	24
Greece	8	-	-	-	-	-	8
Kazakhstan	-	25	-	-	-	-	25
Russia (Amur)	-	22	-	-	-	-	22
Japan	-	8	-	-	-	-	8
Count	***147***	***89***	***102***	***102***	***15***	***25***	***480***
GenBank accession number(s)	***AM156933–AM156934***	***AM156936–AM156943***	***GU134347–GU134358***	***JQ435485–JQ435496***	***No new haplotypes***	***To be provided following GenBank deposition***	
